# Microcapsules and Nanoliposomes Based Strategies to Improve the Stability of Blueberry Anthocyanins

**DOI:** 10.3390/molecules28217344

**Published:** 2023-10-30

**Authors:** Jian Chen, Wenjing Fang, Wei Liu, Jianghua Liu, Pin Gong

**Affiliations:** 1 School of Food Science and Engineering, Shaanxi University of Science and Technology, Xi’an 710021, China; 4268@sust.edu.cn (J.C.);; 2 School of Biological and Pharmaceutical Sciences, Shaanxi University of Science and Technology, Xi’an 710021, China

**Keywords:** anthocyanins, microcapsules, liposomes, stability

## Abstract

Blueberry anthocyanins are water-soluble natural pigments that can be used as both natural antioxidants and natural colorants. However, their structural instability greatly limits their application in the food, pharmaceutical, and cosmetic industries. In this study, blueberry anthocyanin microcapsules (BAM) and blueberry anthocyanin liposomes (BAL) were fabricated based on blueberry anthocyanins. Film dispersion methods were used to prepare the BAL. Their preparation processes were optimized and compared to improve the stability of the blueberry anthocyanins following exposure to light and high temperatures. The BAM were prepared through complex phase emulsification. The blueberry anthocyanins were protected by the shell materials composed of sodium alginate after being formed into BAM. Under the optimal conditions, the embedding rate of BAM and BAL can reach as high as 96.14% and 81.26%, respectively. In addition, the particle size, zeta potential, microtopography, and structure feature information of the BAM and BAL were compared. The average particle sizes of the BAM and BAL were 9.78 μm and 290.2 nm, respectively, measured using a laser particle size analyzer, and the zeta potentials of the BAM and BAL were 34.46 mV and 43.0 mV, respectively. In addition, the optimal preparation processes were determined through single-factor and response surface optimization experiments. The most important factors in the single-factor experiment for the preparation of microcapsules and liposomes were the content of CaCl_2_ and the amount of anthocyanin. The preservation rates in the light and dark were also compared, and the thermal stabilities of the BAM and BAL were characterized through differential thermal scanning. The results showed that both the BAM and BAL maintained the stability of blueberry anthocyanins, and no significant difference was found between the indices used to evaluate their stability. The results of this study provide theoretical support for the development of effective systems to maintain the stability of anthocyanins, thereby improving their bioavailability after ingestion by humans.

## 1. Introduction

Blueberries are rich in many bioactive compounds, such as polyphenols, superoxide dismutase, and vitamin C [1]. Anthocyanins form the main component of the phenolic compounds isolated from blueberries [2]. Our group has previously reported progress on the research of blueberry anthocyanins [3] and has systematically described the biological functions of these anthocyanins. Anthocyanins have properties that can improve health, such as their production of antioxidants; anti-mutagenic, anti-diabetic, anti-obesity, and neuroprotective properties; improvement in vision; and reduction in the risk of coronary heart disease [3].

However, their instability, associated with light, temperature, and pH, greatly limits their use in the food, pharmaceutical, and cosmetic industries [4]. The anthocyanin structure contains many highly unstable phenolic hydroxyl groups, which are highly susceptible to degradation by light, temperature, oxygen, and other factors that are prevalent in the natural environment [5,6]. As described above, the stability of anthocyanins is also related to the pH level because the chemical structure of anthocyanins differs under different pH conditions. Anthocyanins are usually more stable under acidic conditions [2]. In addition, the rate of anthocyanin degradation increases with the increasing temperatures, which affects their stability, as well as, to some extent, their antioxidant activity; therefore, anthocyanins are usually stored at low temperatures [2,7]. Many methods have been explored to improve the stability of anthocyanins, including molecular modification [8], microencapsulation [9], and liposome preparation [10].

Microencapsulation is an important technique as it can improve the stability of anthocyanins. In microencapsulation, different functional materials are encapsulated at the nano and micron scales, which provides the necessary protection for the material that undergoes encapsulation. Such encapsulation can significantly protect the encapsulated material from the external environment and, thus, can prolong its shelf life [11]. Cai et al. [12] used carboxymethyl starch/xanthan gum as the base material for microencapsulation, which improved the stability of blueberry anthocyanins. In vitro release experiments showed that the anthocyanins were retained within the carboxymethyl starch/xanthan gum microcapsules in the stomach and stably released into the intestine [12]. Rosa et al. [13] studied simulated intestinal digestion, which resulted in changes in the loss of anthocyanin from microencapsulated and unencapsulated anthocyanins; their results showed that the encapsulated anthocyanin content was highest in the ileal fraction. In addition, microcapsules fabricated from whey protein and casein [14], gum Arabic [15], and cyclodextrin were effective at maintaining stable blueberry anthocyanins.

Liposomes are artificial membranes in which the hydrophilic head of the phospholipid molecule is inserted into the aqueous phase, and the hydrophobic tail of the liposome reaches into the gaseous phase. Thus, a spherical liposome forms with a double layer of lipid molecules that ranges between 25 and 1000 nm in diameter when stirred [16,17]. Chi et al. [18] and Zhao et al. [19] examined the protective effect of nanoliposomes on anthocyanins. Their results showed that lecithin-cholesterol nanoliposomes could provide additional stability to anthocyanins during storage and simulated gastrointestinal digestion. The protective effect of anthocyanins was tested during simulated digestion in the intestine; the encapsulated anthocyanins decreased to 72.76%, which is a higher level compared to free anthocyanins under the same conditions [18,19]. Changing the levels of anthocyanins used is effective at improving their stability without changing their structures. However, comparative experiments concerning the process and effect of such stability improvements to blueberry anthocyanins using liposome and microencapsulation techniques have not been reported.

In this study, blueberry anthocyanin microcapsules (BAM) and blueberry anthocyanin liposomes (BAL) were prepared and characterized. The two types of anthocyanin used in this study were compared by their particle sizes, appearance, structures, and stability to light and thermal effects. These results provide a theoretical basis and technical support to improve the processing technology of anthocyanins to increase their stability.

## 2. Results and Discussion

### 2.1. Single-Factor Experiment and Response Surface Optimization of the Microcapsule Preparations

#### 2.1.1. Influence of CaCl_2_ Concentration on the BAM Encapsulation Efficiency

**Figure 1:**
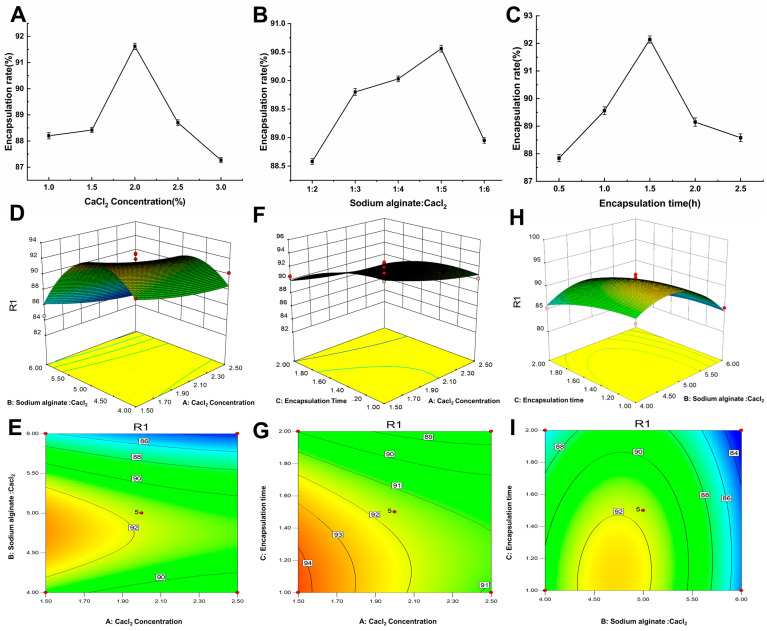
Single-factor and response surface experiments with BAM. (**A**) The effect of CaCl_2_ concentration on encapsulation efficiency. (**B**) The effect of the ratio of sodium alginate to CaCl_2_ on microcapsules. (**C**). The effect of encapsulation time on microcapsules. (**D**–**I**) Three-dimensional response surface and two-dimensional profile of the influence of different factors on the encapsulation efficiency of microcapsules.

The ratio of sodium alginate to CaCl_2_ was 1:3, and the encapsulation time was 1.5 h. Under this condition, the effect of different CaCl_2_ concentrations (1.0%, 1.5%, 2.0%, 2.5%, and 3.0%) on the encapsulation efficiency of the blueberry anthocyanins was examined. Figure 1A shows that the encapsulation effect of the BAM is clearly affected by the concentration of CaCl_2_. The results are consistent with those reported in the literature [20]. At increased concentrations of CaCl_2_, the encapsulation efficiency first increased and then decreased. The highest encapsulation efficiency reached 91.78%. A reason for this could be that when the concentration of CaCl_2_ is too low, it will be difficult for the microcapsule to form because of the soft texture of the microcapsule shell; consequently, anthocyanin may not be embedded successfully, or it can easily leak [21]. Alternatively, when the CaCl_2_ concentration is too high, the formed microcapsules are sticky and have irregular shapes in the chitosan/alginate polyelectrolyte complexes [22]. Because the collision between microcapsules first forms during the fabrication process—instead of the curing reaction—the capsules rupture, thus greatly reducing the encapsulation efficiency of the microcapsules. Therefore, the encapsulation was most effective at a concentration of 2.0% CaCl_2_.

#### 2.1.2. Effect of the Ratio of Sodium Alginate to CaCl_2_ on the BAM Encapsulation Efficiency

A CaCl_2_ concentration of 2.0% was selected, and the encapsulation time was 1.5 h. Under this condition, the effects of different ratios of sodium alginate and CaCl_2_ (1:2, 1:3, 1:4, 1:5, and 1:6) on the encapsulation efficiency of the blueberry anthocyanins were examined. Figure 1B shows that with the increasing proportion of CaCl_2_, the encapsulation rate increased slowly and then decreased rapidly. At a ratio of sodium alginate to CaCl_2_ of 1:5, the encapsulation rate reached its maximum of 91.78%. Under certain conditions, a higher mass fraction of sodium alginate increases the solidification rate [23]. When the ratio of CaCl_2_ is insufficient, the degree of solidification of the microcapsules is also insufficient, and their outer shell is soft. This leads to uneven and irregular formation, serious microcapsule deformation, and eventually a poor encapsulation effect [24]. Alternatively, at a high ratio of CaCl_2_, an excessive amount of calcium alginate formed with a dense shell, which not only affects the freeze-drying efficiency of the microcapsules, but also reduces their encapsulation effect. Torre et al. [25] showed that an excessively high sodium alginate content will reduce the embedding rate, which may be related to the shrinkage of the beads during the gelation process.

#### 2.1.3. Effect of Encapsulation Time on the Rate of BAM Encapsulation

A CaCl_2_ concentration of 2.0% was selected, and the ratio of sodium alginate to CaCl_2_ was 1:5. Under this condition, the effects of different encapsulation times (0.5, 1.0, 1.5, 2.0, and 2.5 h) on the encapsulation efficiency of the BAM were examined. Figure 1C shows that when the encapsulation time is within a range of 1.0–2.5 h, with the extension of time, the encapsulation rate increased firstly and then decreased slowly. At an encapsulation time of 1.5 h, the microencapsulation efficiency reached its maximum value of 92.37%. When there was a short encapsulation time, the contact between most anthocyanin emulsions and the sodium alginate emulsion was insufficient; the oil-in-water system was unstable, and most of the anthocyanins were still free in the mixture of emulsion and sodium alginate [26]. Therefore, only a few microcapsules formed. However, when the encapsulation time is too long, a stable oil-in-water system forms from the anthocyanin emulsion and sodium alginate. Continuous stirring will cause the formed microcapsules to rupture, which results in anthocyanin leakage. This will eventually lead to a decrease in the encapsulation rate of the microcapsules. 

#### 2.1.4. Response Surface Model and Significance Test

Based on the preliminary screening results of the effect of the various processing parameters on the preparation of the microcapsules according to the single-factor experiment, three major factors that had a significant effect on the preparation of the microcapsules were selected for further study. These included the concentration of CaCl_2_ (X_1_), the ratio of sodium alginate to CaCl_2_ (X_2_), and the encapsulation time (X_3_). Each factor was set at three levels. 

The Box-Behnken experimental design principle was applied using the Design Expert software (Ver. 10.0, Stat-Ease). The encapsulation rate was taken as the response value; 17 groups of preparation prescriptions were designed, and the results of the response surface scheme and response value are shown in Table 1.

The data shown in Table 1 were subjected to multiple regression fitting. A quadratic polynomial regression model of the encapsulation rate and independent variables was obtained. The regression equation is as follows:

Y = 91.60 − 1.24X_1_ − 2.39X_2_ − 1.70X_3_ − 0.12X_1_X_2_ + 0.5X_1_X_3_ + 0.48X_2_X_3_ + 0.41X_12_ − 4.73X_22_ − 1.09X_32_

A total of three factors—namely CaCl_2_ concentration (X1), sodium alginate to CaCl_2_ ratio (X2), and encapsulation time (X3)—were examined in this study. The response value of the embedding efficiency (Y) was used to optimize the preparation process of the BAM according to the experimental Box-Behnken design principle. The results of the analysis of variance for each factor are shown in Table 2. The quadratic terms of the content of the CaCl_2_, the ratio of sodium alginate to CaCl_2_, and the encapsulation time were extremely significant. The *p*-values can be used to gauge the extent to which the encapsulation rate of the BAM is affected by a single factor. According to Table 2, the order was X_1_ > X_3_ > X_2_. This indicated that the degree of influence of the three factors on the encapsulation rate of the BAM was the concentration of CaCl_2_ > encapsulation time > ratio of sodium alginate to CaCl_2_. The *p*-values of the three quadratic terms were all <0.05, indicating that the quadratic terms all reached significant levels for the encapsulation efficiency of the blueberry anthocyanins. The quadratic term model of the regression model was extremely significant (*p* < 0.01), and the lack of fit term was not significant (*p* = 0.1905). These values indicated that this equation is a good fit for the experimental data within the scope of the experiment. The model selection is moderate, and the experimental results are highly reliable. Therefore, this model is highly practical for predicting the encapsulation efficiency of BAM.

#### 2.1.5. Response Surface Analysis of the Encapsulation Efficiency of Microcapsules

**Figure 2:**
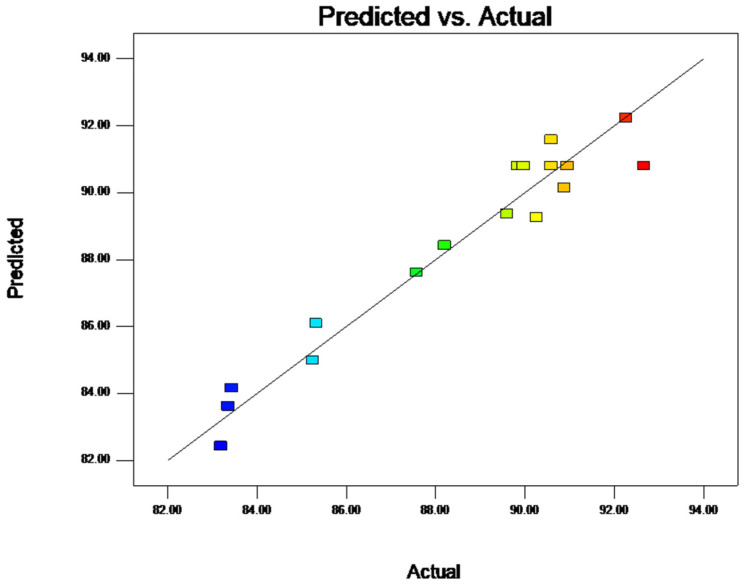
Straight distribution of actual value and prediction fit for BAM. Note: The various hues of the squares correspond to the encapsulation rate of each experiment.

Figure 2 shows the distribution plot of the predicted amounts versus the actual amounts for the encapsulation of BAM. When the points are closer to the 45 degree line, this indicates that there are better estimations of the RSM model. Based on this plot, the model could appropriately fit the data.

A three-dimensional response surface diagram of the regression model was obtained according to the regression equation analysis using the Design-Expert software. Figure 1D–I shows the response surface and contour map of the influence of the three factors of the CaCl_2_ concentration, ratio of sodium alginate to CaCl_2_, and encapsulation time on the encapsulation efficiency of the blueberry anthocyanins. The steeper slope of the response surface suggests that the interaction between the two factors increases. A darker response surface color indicates that the influencing factors exert a significant effect on the experimental results. A lighter response surface color indicates that the influencing factors have a weak effect on the experimental results. As the degree of contour ellipse deepens in color, the two factors of interaction have a more significant effect on the experimental results. In addition, the maximum value is usually part of a closed ellipse or circle.

As shown in Figure 1, the concentration of the CaCl_2_ has the most significant effect on the encapsulation efficiency of the microcapsules. As the concentration of CaCl_2_ increased, the encapsulation efficiency of the BAM gradually increased (Figure 1D,E). At a CaCl_2_ concentration of 2.0%, the blueberry flower encapsulation efficiency of the penicillin microcapsules was the largest. In contrast, the influences of both the ratio of CaCl_2_ to sodium alginate and the encapsulation time were weaker. The reason for this is that when the concentration of CaCl_2_ and the ratio of CaCl_2_ to sodium alginate increase, the slope becomes increasingly moderate, as shown in the contour map of the interaction between the CaCl_2_ concentration and the ratio of CaCl_2_ to sodium alginate (Figure 1F,G). In addition, with the increasing ratio of CaCl_2_ to sodium alginate and the prolonged encapsulation time, the response surface showed no apparent increase. Compared with the other two graphs, the slope in this graph was the lowest (Figure 1H,I). The ratio of sodium alginate to CaCl_2_ had the least effect on the encapsulation efficiency of the microcapsules. Therefore, the importance of each factor for the response value can be ordered as follows: content of CaCl_2_ > encapsulation time > ratio of sodium alginate to CaCl_2_.

#### 2.1.6. Parameter Optimization and Verification

The encapsulation rate of the BAM shows some correlation with each of the above three factors. To maximize the encapsulation efficiency, it is necessary to comprehensively consider all of the factors. According to the Box-Behnken design model analysis, the optimal preparation conditions for BAM were as follows: a CaCl_2_ concentration of 2.0%, a ratio of sodium alginate to CaCl_2_ of 1:5, and encapsulation time of 1.5 h. To further improve the reliability of the model, the anthocyanin microcapsules were prepared three times under these optimal conditions, and the encapsulation efficiencies were determined separately. The results show that the theoretical value of the BAM encapsulation rate is 92.75%. The final three experimental values of the encapsulation efficiency were 91.56%, 92.97%, and 91.88% under optimal conditions, and the average value was 92.13% with a theoretical value error < 5%. This result indicates that the preparation process optimized by the response surface method is highly practical and provides an accurate reference value.

### 2.2. Single Factor Experiment and Response Surface Optimization of the BAL Preparation

#### 2.2.1. Effect of the Amount of Anthocyanins Added on Encapsulation Efficiency

**Figure 3:**
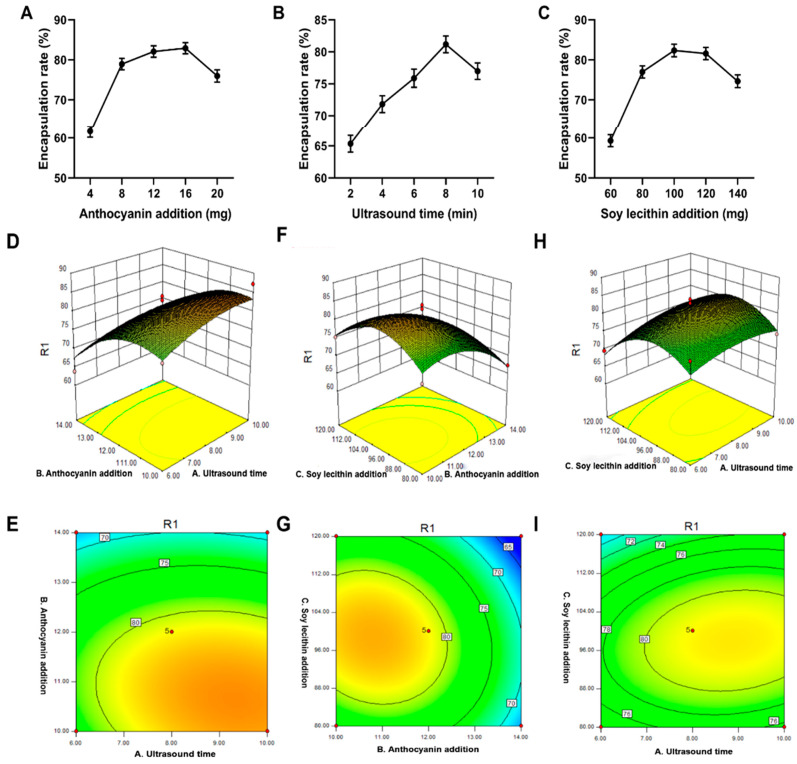
Univariate and response surface experiments with BAL. (**A**) The effect of the amount of anthocyanins added on encapsulation efficiency. (**B**) The effect of ultrasonic time on encapsulation efficiency. (**C**) The effect of lecithin addition on encapsulation efficiency. (**D**–**I**) 3-D response surface plots and 2-D contour plots showing effects of various factors. 2-D, two-dimensional; 3-D, three-dimensional; BAL, blueberry anthocyanin liposomes.

At an ultrasonication time of 12 min, 4, 8, 12, 16, and 20 mg of blueberry anthocyanins were added separately to 100 mg of soybean lecithin. As shown in Figure 3A, the BAL encapsulation efficiency increases with the increasing addition of blueberry anthocyanins. When 16 mg of blueberry anthocyanins were added, the encapsulation rate reached 82.94%. After this, the encapsulation rate declined slowly. There may be two reasons for this response. First, the interaction between the anthocyanin cation and the negatively charged phospholipid is enhanced [27]; the hydrophobic part of the anthocyanin interacts with the fatty chain of the phospholipid, and part of the anthocyanin is distributed into the bilayer membrane [28]. Alternatively, an excessive amount of anthocyanin in the liposomes could possibly reduce the surface charge and electrostatic repulsion between the vesicles, thus decreasing the encapsulation efficiency [29,30].

#### 2.2.2. Effect of Ultrasonic Time on the Encapsulation Rate

When 100 mg of soybean lecithin and 12 mg of blueberry anthocyanin were added, ultrasonication times of 2, 4, 6, 8, and 10 min were tested separately. As shown in Figure 3B, the encapsulation efficiency of the liposomes increased with the increasing ultrasonication time and began to decrease after 8 min. If the ultrasonication time is too short, the film formation may become uniform. Therefore, the liposomes will rupture; the anthocyanins will leak, and the liposome encapsulation efficiency will be reduced [31]. In contrast, a long ultrasonic time will lead to a rapid increase in temperature. The increase in energy input will cause high temperatures that result in the degradation of anthocyanins because of the disruption of the van der Waals effect between the phospholipid molecules [32]. Consequently, the fluidity of the phospholipid bilayer was improved. In addition, the increased energy input may also partly disrupt the interaction between the anthocyanins and phospholipids or cholesterol, ultimately leading to a decrease in the efficiency of liposome encapsulation. This result matches those of Liu et al., who suggested that the appropriate ultrasonication time can increase the stability of liposome suspension [33]. Therefore, the best ultrasonication time is 8 min because the liposome encapsulation rate reached 81.68% after this time.

#### 2.2.3. Effect of Soy Lecithin on Encapsulation Efficiency

At an added amount of blueberry anthocyanin of 12 mg and an ultrasonication time of 12 min, 60, 80, 100, 120, and 140 mg of soybean lecithin were added. As shown in Figure 3C, the encapsulation efficiency of the liposomes increases with the increasing levels of soybean lecithin. The maximum value was 82.31%, which was obtained when the blueberry anthocyanin was added to 100 mg of soybean lecithin. After this, the value started to slowly decline. This result may be due to the negative charge of soybean lecithin itself, which is saturated with the cations of anthocyanins; the excess negative charge disrupts the stability of the liposomes and causes a decrease in the encapsulation rate [34]. The other reason is that with the increasing amounts of soybean lecithin, the anthocyanins are distributed to the liposome bilayer membrane; with the increasing distribution of anthocyanins, the original arrangement order of the phospholipids in the bilayer membrane may be disrupted [34]. This disruption renders the whole liposome system unstable, and the number of bilayer lipids decreases accordingly. Finally, the encapsulation rate decreases. 

#### 2.2.4. Response Surface Model and Significance Test

Based on the preliminary experiments, single-factor experiments were used to investigate the effects of three factors, including the amount of anthocyanins, the ultrasonic time, and the addition of soy lecithin. Each experiment was repeated three times to determine the following factors as the fixed value: a total of 15 mg of anthocyanins were added, the ultrasonic time was 8 min, and 100 mg of soy lecithin was added.

Secondly, according to the single factor experiment, three major factors that have a significant effect on the preparation of liposomes were selected as the object of investigation. These included the amount of anthocyanins (X_1_), the ultrasonic time (X_2_), and the addition of soy lecithin (X_3_). Each factor was set at three levels.

The encapsulation rate was taken as the response value according to the Box-Behnken experimental design principle in the Design Expert software. The results of the response surface scheme and response value are shown in Table 3.

Multiple regression fitting was performed on the data shown in Table 3 to obtain a quadratic polynomial regression model of the encapsulation rate and independent variables. The regression equation was as follows:

Y = 81.27 + 1.76X_4_ − 6.13X_5_ − 1.70X_6_ − 1.14X_4_X_5_ + X_4_X_6_ − 1.52X_5_X_6_ − 1.91X_45_ − 5.06X_55_ − 6.43X_65_

A total of three factors, including the amount of anthocyanins added (X_4_), the sonication time (X_5_), and soy lecithin addition (X_6_), were selected. The response value of the embedding efficiency (Y) was used to optimize the process of preparing the blueberry anthocyanin liposomes according to the experimental Box-Behnken design principle. The results of the analysis of variance for each factor are shown in Table 4. The secondary terms of the ultrasonication time, the amount of blueberry anthocyanins, and the amount of soybean lecithin are significant. The *p*-value can be used to assess the extent to which the encapsulation efficiency of the BAL is affected by a single factor. The result shown in Table 4 can be ordered according to X_2_ > X_1_ > X_3_. This result means that the degree of influence of the three factors on the BAL encapsulation efficiency can be ordered according to the amount of blueberry anthocyanins > ultrasonication time > amount of soybean lecithin. The *p*-values of the three quadratic terms were all < 0.05, indicating that the quadratic terms all reached significant levels for the encapsulation efficiency of blueberry anthocyanin. The quadratic term model of the regression model was extremely significant (*p* < 0.01), and the lack-of-fit term was not significant (*p* = 0.0787), indicating that this equation fits the experiment well. The model selection is moderate, and the experimental results are highly reliable. Therefore, this model is highly practical for predicting the encapsulation efficiency of BAL.

#### 2.2.5. Response Surface Analysis of Encapsulation Efficiency

**Figure 4:**
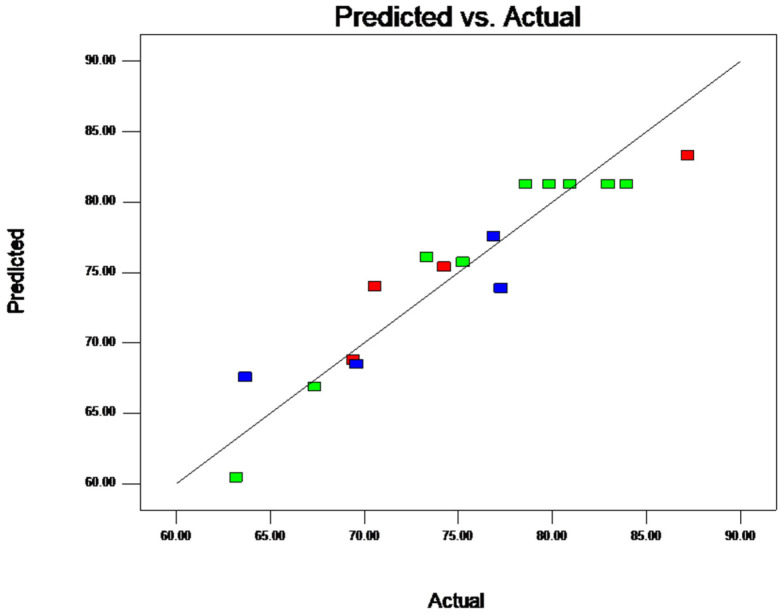
Straight distribution of actual value and prediction fit for BAL. Note: The various hues of the squares correspond to the encapsulation rate of each experiment.

Based on the actual value and predicted fitting line distribution, as shown in Figure 4, the model shows that when the actual values and predicted values were closer on a straight line, the model exhibited a higher degree of fit. This indicates that the experimental data were accurate and reliable. Thus, the model is suitable to simulate the experimental data.

A three-dimensional response surface diagram of the regression model was obtained according to the regression equation analysis conducted using the Design-Expert software. Figure 3D–I shows the interaction of the amount of blueberry anthocyanins, the ultrasonication time, and the amount of soybean lecithin on the BAM encapsulation efficiency.

An analysis of the response surface diagram shows that the addition of blueberry anthocyanins had the most significant effect on the efficiency of the liposome encapsulation. Furthermore, the efficiency of the BAL encapsulation increased with the addition of increasing amounts of blueberry anthocyanins. When the addition of these anthocyanins increased to 12 mg, the efficiency of the BAL encapsulation reached its maximum (Figure 3D). After that, the response surface became steeper, and the efficiency of BAL encapsulation significantly decreased. The effect of the ultrasonication time on the efficiency of encapsulating blueberry anthocyanin liposomes was not as significant as the effect of the blueberry anthocyanin on the encapsulation efficiency (Figure 3E). In addition, the addition of soybean lecithin had the smallest effect on the efficiency of BAL encapsulation. Figure 3I shows that the response surface did not exhibit a clear trend of increasing values as the amount of soybean lecithin increased. The significance of each factor to the response value was as follows: amount of blueberry anthocyanins > ultrasonication time > amount of soybean lecithin.

#### 2.2.6. Parameter Optimization and Verification

The BAL encapsulation rate correlated with each of the above three factors to some degree. To maximize the encapsulation efficiency, it is necessary to comprehensively consider all of the factors. According to the Box-Behnken design model analysis, the optimal preparation conditions for BAL were as follows: 12 mg of blueberry anthocyanin, an ultrasonication time of 12 min, and 100 mg of soybean lecithin. To further improve the reliability of the model, anthocyanin liposomes were prepared three times under optimal conditions. The calculated theoretical value of the blueberry anthocyanin liposome encapsulation efficiency was 81.26%. The final experimental values of the liposome encapsulation efficiencies were 81.56%, 80.67%, and 81.61% under optimal conditions, and the average value was 81.28% with a theoretical error < 5%. This result proves that the preparation process optimized by the response surface method is highly practical and accurate and can serve as a reference value.

### 2.3. Quality Evaluation of BAM and BAL

#### 2.3.1. Particle Size and Zeta Potential

**Figure 5:**
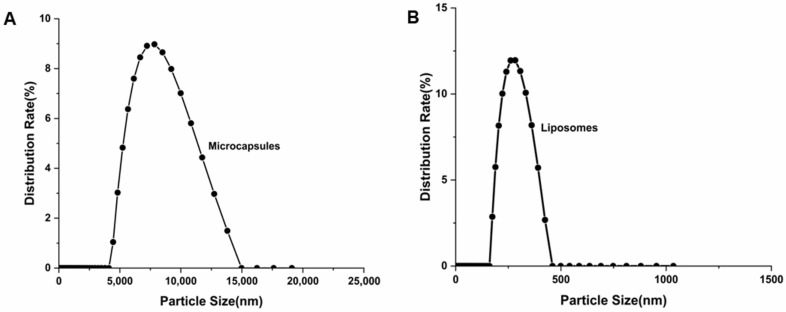
Particle size distribution diagram of (**A**) BAM and (**B**) liposomes.

Figure 5A,B shows that the average particle size of the BAM was 9.78 μm, and that of the zeta potential was −34.46 mV. The average particle size of the BAL was 290.2 nm, and the zeta potential was −43.0 mV. The particles of the BAM were much larger than those of the BAL, and the BAL were monodispersed and had a more uniform distribution of particle sizes than the BAM. Moreover, the absolute value of the zeta potential of the BAM and BAL prepared in the experiment exceeded 30 mV, indicating that after process optimization, the BAM and BAL were highly stable. The zeta potential represents the surface charge of nanoparticles, which determines the repulsion between charged nanoparticles, and thus affects their storage stability [32]. Particles with an absolute zeta potential exceeding 30 mV have relatively high repulsive interactions and are therefore considered stable. Although these two kinds of micromaterials show little difference in stability, their particle sizes will impact the subsequent application of anthocyanins. BAL have a smaller particle size, and thus a wider range of applications. However, the large particle size of BAM can encapsulate the compounds more efficiently and avoid wasting the anthocyanins [35].

#### 2.3.2. FT-IR Analysis

**Figure 6:**
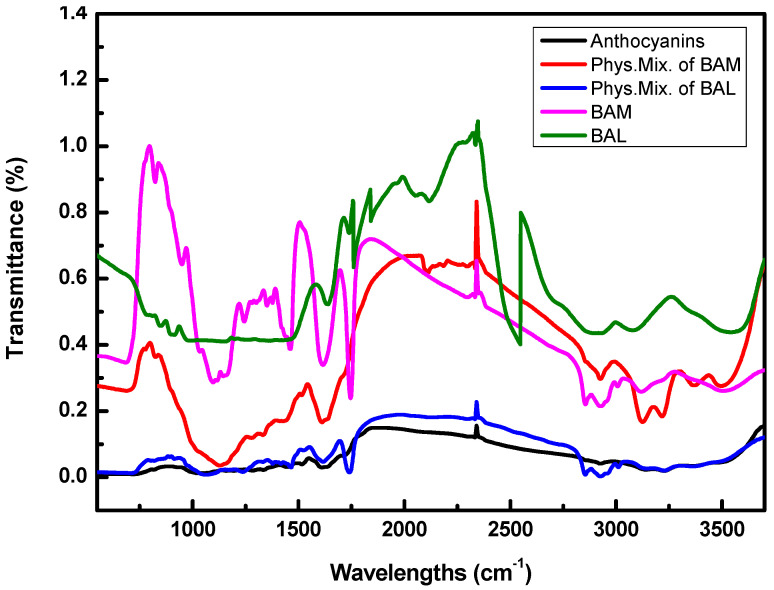
FT-IR spectra of the blueberry anthocyanins, the physical mixture of the blueberry anth-cyanins and raw materials for making (Phys.Mix. Of BAM), the physical mixture of the blueberry anthocyanins and raw materials for making BAL (Phys.Mix. Of BAL), BAM and BAL.

Fourier-transform infrared spectroscopy (FT-IR) analysis can be used to explore whether the new phase of BAM is formed. Figure 6 lists the infrared spectra of the blueberry anthocyanins, the physical mixture of blueberry anthocyanins and raw materials for making BAM, the physical mixture of blueberry anthocyanins and raw materials for making BAL, as well the BAM and BAL.

In the anthocyanin spectrum, the peaks at 1612 cm^−1^ and 1517 cm^−1^ correspond to the benzene ring structure of anthocyanins. The peaks observed at 821 cm^−1^, 776 cm^−1^, and 668 cm^−1^ represent the out-of-plane bending vibrations of the CH groups in the benzene ring of anthocyanins. The absorption peaks at 3237 cm^−1^ and 3221 cm^−1^ correspond to the stretching vibration of -OH, which is present in the phenolic hydroxyl group of anthocyanin, the alcoholic hydroxyl group of anthocyanin sugar moiety, water molecules, and hydrogen bonds.

For BAM, the characteristic peaks of sodium alginate at 2933 cm^−1^ and 1604 cm^−1^ corresponded to the C-H and C=O contraction vibrations, respectively. The absorption peaks at 1618 cm^−1^ and 1413 cm^−1^ corresponded to the C-O bond contraction vibrations. Compared with the physical mixture of BAM materials, there was a noticeable change in the infrared spectrum of the BAM within the range of 2800–3300 cm^−1^. This change suggests that there was a slight shift in the C-H bond and C=O characteristic peak of sodium alginate due to its solidification by CaCl_2_ for forming the shell structure of the microcapsules. A further analysis showed that the infrared spectrum of the BAM has a weakened characteristic peak at 3237 cm^−1^, which fully indicates that blueberry anthocyanins had been successfully embedded. For the BAL, the symmetrical vibration of P=O was located at 1239 cm^−1^, and the stretching vibration of C=O was located at 1750 cm^−1^, which is considered to be the absorption peak of soybean lecithin. The characteristic anthocyanin peak in the infrared spectrum of liposome anthocyanin disappeared at 3221 cm^−1^, while it was still observed in the physical mixture of the BAL at 3221 cm^−1^. This result indicates that blueberry anthocyanins have entered the liposome, showing that the BAL were successfully prepared. In addition, the characteristic peak of soybean lecithin at 1751 cm^−1^ in the BAL shifted compared with the physical mixture of the BAM. The reason for this may be that the soybean lecithin reacted when the anthocyanins were encapsulated, which caused a shift in the absorption peak. The spectra shifting of the BAM and BAL within the characteristic absorption peak range of anthocyanidins suggests that both BAM and BAL are not simply a combination of raw material and anthocyanidins, but indicates an interaction between these components.

#### 2.3.3. SEM Characterization

**Figure 7:**
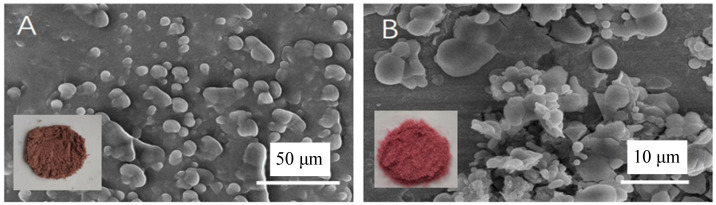
SEM image of blueberry anthocyanin (**A**) microcapsules and (**B**) liposomes (650-fold).

The microscopic morphology of the BAM and BAL were characterized through scanning electron microscopy (SEM), and the illustrations are photographs of their powder. Figure 7A shows that most of the microcapsules are spherical and have relatively smooth surfaces without cracks or breaks, but some microcapsules exhibited a surface depression. This depression may be due to the uneven wrinkles of the wall material caused by the rapid water loss during the freeze-drying process [36]. In Figure 7B, spherical or oval liposomes can be observed. The surface of the liposome samples is relatively smooth, and some liposomes show irregular shapes and aggregation, which may be due to the collapse of liposome vesicles before sample treatment. This may have resulted in varying degrees of changes in their morphology and particle size, which ultimately led to the appearance of liposomes with irregular shapes.

#### 2.3.4. Light Stability

The anthocyanins in blueberry have antioxidant and antibacterial properties. Thus, improving their light and thermal stabilities can increase their retention rate and reduce losses, thereby reforming their antioxidant effects in food and cosmetics.

**Figure 8:**
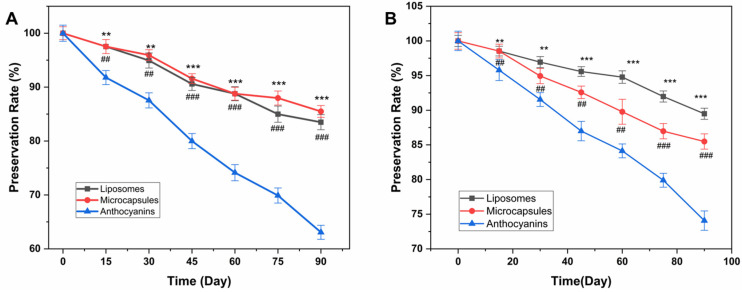
Preservation rate of BAM and liposomes (**A**) light-proof condition and (**B**) under light. *p* < 0.01 **; *p* < 0.001 ***. *p* < 0.01 ^##^; *p* < 0.001 ^###^.

Figure 8A shows the preservation rates of the blueberry anthocyanins, BAM, and BAL under light conditions, while Figure 8B shows these data under dark conditions. In the light, the BAM and BAL have clear differential effects on the anthocyanins, but the BAM and BAL have no apparent changes. The BAM and BAL significantly improved the stability of the anthocyanins in the light over time.

The preservation rates of the BAM, BAL, and anthocyanins decreased by 8.27%, 10.12%, and 23.13%, respectively, in the dark at 25 °C. Under natural light, these preservation rates decreased to 10.42%, 16.52%, and 37.33%, respectively. Light can disrupt the molecular structure of blueberry anthocyanins. Although their imbedding as BAM or BAL cannot guarantee that the anthocyanins will not be degraded at all, their embedding has some effect on the heat and oxygen insulation of the anthocyanins. The final content of anthocyanin in BAM is always higher than that of untreated anthocyanin.

Therefore, BAM and BAL can improve the stability of anthocyanin storage in the light. The protective effect of BAM may be due to the microcapsule shell formed by calcium alginate, which acts as a physical barrier, and thus obstructs the degradation of anthocyanins by factors such as light and oxygen. The protective effect of BAL may be reflected in the following two aspects. The outer membrane of the liposome can act as a physical barrier, and thus obstructs the degradation of anthocyanins by factors such as light and oxygen. Alternatively, soybean lecithin can prevent anthocyanins from being oxidized at the expense of its own oxidation, and cholesterol can prevent the lipids in BAL from being oxidized. Consequently, the possibility of the anthocyanins being oxidized is greatly reduced. This reasoning suggests that BAL can effectively improve the stability of anthocyanins and ensure that their preservation rate is controlled within an acceptable range. These results indicate that as time goes by, the content of anthocyanins and their liposomes decreases, and the decline of unembedded anthocyanins becomes more apparent [37,38]. Our results are similar to reference [39], in which the carriers, maltodextrin, pectin, and soy protein isolate were able to provide protection to monomeric anthocyanin and antioxidant activity higher than 76% and 73%, respectively, after 90 days under UV light.

#### 2.3.5. Thermal Stability

**Figure 9:**
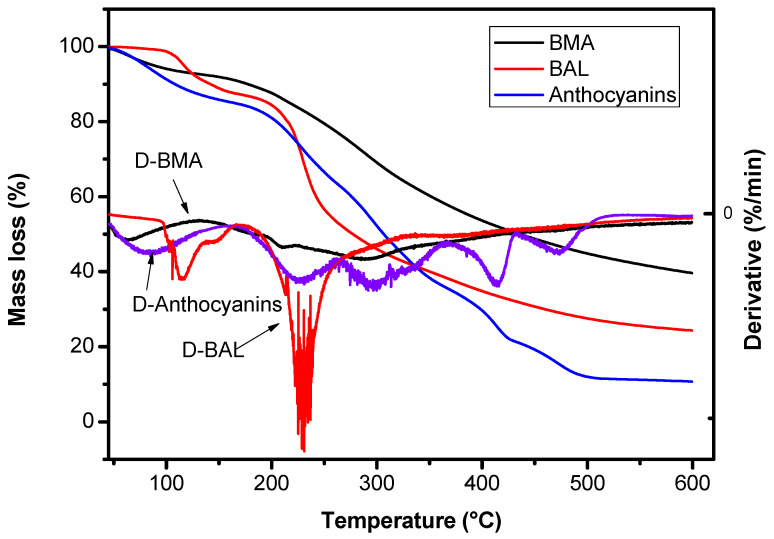
TGA curves and DTG curves of anthocyanins, BAM and BAL.

Thermogravimetric Analysis (TGA) can reflect the variation in sample quality with the temperature under program controlled temperature, and can be used to evaluate the thermal stability of BAM, BAL, and anthocyanins. The TGA and DTG curves of anthocyanins, BAM, and BAL are presented in Figure 9. It is evident that within the range of 30–600 °C, the mass loss rates of the BAM and BAL were lower compared to that of anthocyanins. As can be seen, the anthocyanins exhibited an initial mass loss at approximately 80 °C, followed by a rapid decline at around 200 °C. In contrast, significant changes in the BAL were not observed until the temperature reached 114 °C, and thermal decomposition of the wall material occurred between temperatures 210–285 °C. This observation can be attributed to the denaturation of the soy lecithin present as a component in the wall material. Under high temperatures, the soy lecithin underwent structural rearrangement, from an ordered crystal structure to a disordered one, through endothermic reactions. In addition, the slight mass loss of the BAM appeared around 300 °C, which is mainly caused by the decomposition of calcium and sodium salts. Comparatively, the BAM demonstrated better thermal stability than the BAL due to their denser shell structure formed by curing sodium alginate with CaCl_2_, which provided enhanced protection for anthocyanins against heat damage. Consequently, more heat is required to disrupt this protective structure, resulting in higher thermal stability for BAM when compared with both anthocyanins and BAL. This suggests that the thermal stability of anthocyanins could be improved by the microencapsulation treatment and liposomalization process.

## 3. Materials and Methods

### 3.1. Materials

Blueberry anthocyanins were purchased from Xi’an Shengqing Biological Technology Co., Ltd. (Xi’an, China), with a purity of 25% of the anthocyanins. Corn oil was purchased from Shan Dong Xiwang Food Co., Ltd. (Leling, China). Span-80 and Tween-80 were purchased from Sinopharm Chemical Reagent Co., Ltd. (Shanghai, China). Sources of absolute ethanol and calcium chloride were purchased from Shantou Guanghua Chemical Factory (Shantou, China). Sodium alginate was purchased from Shanghai Lanji Technology Development Co., Ltd. (Shanghai, China). Anhydrous citric acid was purchased from Shanghai Shanpu Chemical Co., Ltd. (Shanghai, China). Soybean lecithin and cholesterol were purchased from Chengdu Jinshan Chemical Reagent Co., Ltd. (Chengdu, China). 3-*O*-glucoside was purchased from Shanghai Yuanye Biotechnology Company (Shanghai, China). Potassium bromide was purchased from Tianjin Kemeou Chemical Reagent Co., Ltd. (Tianjin, China).

### 3.2. Preparation of BAM and Single-Factor Experiments

The sodium alginate is inherently safe, non-toxic, and innocuous, exhibiting excellent biocompatibility. It serves as a commonly employed material for the preparation of microcapsules [40]. Sodium alginate was used as the wall material; blueberry anthocyanins were used as the core material; Span-80 and Tween-80 were used as emulsifiers; absolute ethanol was used as the emulsifier; and CaCl_2_ was used as the curing agent. Solutions of CaCl_2_ with different concentrations were prepared, and the volume ratio of CaCl_2_ to sodium alginate was 1:3. First, the sodium alginate was heated in a water bath at 85 °C for 20 min until it had completely dissolved. It was then cooled to room temperature for later use. Moreover, the prepared blueberry anthocyanin solution, the emulsifiers Span-80, Tween-80, and ethanol were placed in a magnetic stirrer and mixed for 1 h to obtain the blueberry anthocyanin emulsion. Secondly, the blueberry anthocyanin emulsion was slowly added to the sodium alginate and mixed using a magnetic stirrer for 1.5 h to obtain uncured blueberry anthocyanin microcapsules. Third, these uncured blueberry anthocyanin microcapsules were slowly added to the CaCl_2_ using the atomization method. After solidification, the mixture was centrifuged at 4000 rpm for 15 min, after which the microcapsules had been deposited on the lowest layer. Fourth, blueberry cyanine vegetarian microcapsule powder was obtained through freeze-drying for 48 h at a vacuum degree of 0.09 MPa and a temperature of −50 °C. The effects of the encapsulation time, the ratio of sodium alginate to CaCl_2_, and the concentration of CaCl_2_ on the encapsulation rate were studied.

Based on the results of the single-factor experiments, optimization was performed according to the experimental protocol design shown in Table 5. A total of 17 sets of experimental conditions with three factors and three levels were automatically generated using the Design Expert software (Stat-Ease, Inc., Minneapolis, MN, USA). 

### 3.3. Preparation of BAL and Single Factor Experiments

BAL were prepared using the film-ultrasonic dispersion method. A total of 12 mg of blueberry anthocyanins, 100 mg of soy lecithin, and 5 mg of cholesterol were placed in a round bottom flask. A volume of 60 mL of absolute ethanol was added until all the ingredients had dissolved. A rotary evaporator was used to concentrate the mixture at 45 °C under reduced pressure for 15 min until the absolute ethanol was completely removed. After that, a lavender uniform film formed on the inner wall of the round bottom flask. A volume of 50 mL of a 3% glucose solution was then added to the bottle, and the mixture was rotated and heated at 45 °C for 30 min until the film on the inner wall of the round-bottom flask completely peeled off and dissolved in the glucose solution. After ultrasonication of the suspension for 10 min, a uniform suspension was obtained. Finally, a 0.45 μm microporous membrane was used to filter and obtain the liposomes. The liposome powder was obtained by freeze-drying the liposomes through vacuum drying for 48 h at a vacuum degree of 0.09 MPa and a temperature of −50 °C. When utilized as an indicator of blueberry anthocyanins, the effects of the amount of anthocyanin added, the time of ultrasonication, and the amount of soybean lecithin added for the liposomerization of the blueberry anthocyanins on the encapsulation rate were examined.

Based on the single-factor experiment, the optimization process was conducted according to the experimental protocol design shown in Table 6. A total of 17 sets of experimental conditions with three factors and three levels were automatically generated using the software.

### 3.4. Determination of Blueberry Anthocyanin Contents in BAM and BAL

The content of blueberry anthocyanins was determined through pH differential spectrophotometry [41,42]. A total of 4 mL of buffer at pH 1.0 (0.5 mol/L Ca/HCl) and pH 4.5 (0.5 mol/L CA/dipotassium phosphate) was added to 1 mL of the anthocyanin solution. The solutions were then stored in the dark for 60 min, and the absorbance of the samples was measured at 520 nm and 700 nm. The following Formulae (1) and (2) were used to calculate the anthocyanin content:

(1) $$Anthocyanin\;content=\left(A\times Mw\times DF\times 1000\right)/\left(\varepsilon \times L\right)$$

(2) $$A=\left(A_{520}-A_{700}\right)pH1.0-\left(A_{520}-A_{700}\right)pH4.5$$

where *DF* is the dilution time; *Mw* is the molar mass (449.2 g/moL); *ε* is the molar extinction coefficient of cyanidin-3-*O*-glucoside (26,900 L/moL/cm); and *L* is the cuvette path length (1 cm).

### 3.5. Determination of the Encapsulation Efficiency

#### 3.5.1. Determination of the BAM Encapsulation Efficiency

Blueberry anthocyanin microcapsules (0.2 g) were rinsed repeatedly using double distilled water. After the surface of the blueberry anthocyanins had been rinsed completely, the solution was collected and subjected to volumetry using a 50 mL volumetric flask. The anthocyanin content in the solution was measured, as described in Section 3.4. As a result, the content measured above presents the content of anthocyanins on the surface that were not embedded in the microcapsule. Similarly, 0.2 g of BAM was weighed and ultrasonicated in citric acid for 15 min. After full dissolution, the samples were centrifuged at 5000 rpm for 5 min and subjected to volumetry using a 50 mL volumetric flask. The total anthocyanin amount of the BAM was then measured, as described in Section 3.4. Finally, the blueberry anthocyanin encapsulation efficiency can be calculated using Formula (3).

Encapsulation rate = (Total anthocyanin content − Unembedded anthocyanin content)/Total anthocyanin content × 100%(3)

#### 3.5.2. Determination of the BAL Encapsulation Efficiency

The BAL preparation was centrifuged at 4 °C and 12,000 rpm for 30 min. The deposited liposomes were then washed twice with 1 mL sodium citrate buffer (pH = 3.5). After further centrifugation for 30 min, the purple upper phase solution was separated and diluted with sodium citrate buffer (1:1) to obtain free anthocyanins. Moreover, 1% of HCl (*w*/*v*) was added. After stirring for 1 min, the suspension was centrifuged at 4 °C and 12,000 rpm for 30 min. The purple upper phase solution that contained the total mass of anthocyanins was then separated. The amount of anthocyanins was determined as described in Section 3.4. Finally, the encapsulation efficiency of BAL was calculated using Formula (4):

Encapsulation efficiency of liposome = (Total anthocyanins − Free anthocyanins)/total anthocyanins × 100%(4)

### 3.6. Characterization and Evaluation of Stability

#### 3.6.1. Characterization

The average particle size and zeta potential of the BAM and BAL were measured using a laser particle size analyzer (BOS-1076, Xiamen Boshi testing Equipment Co., LTD, Xiamen, China) in DLS mode. A laser with a wavelength of 535 nm and a scattering angle of 173° was used to measure the samples at 25 °C. Each sample was measured three times. SEM (Apreo 2, Thermo Fisher Scientific, Waltham, MA, USA) was used to observe the surface morphology of the samples under an accelerating voltage of 10 kV. FT-IR (VERTE 70, Bruker, Bremen, Germany) was used to monitor the functional groups of blueberry anthocyanins, BAM, and BAL with a wave number range of 4000–400 cm^−1^ and a resolution of 16 cm^−1^.

#### 3.6.2. Light and Thermal Stability

Stability in the light was evaluated using the following method: anthocyanin samples, BAM powder, and BAL powder were placed at 25 °C for 90 d under both natural light and in the dark. The anthocyanin content of the samples was measured every 10 d. The thermal stability was evaluated using the following method: a total of 5 mg of blueberry anthocyanin, BAM, and BAL were plated in the crucible and then subjected to TGA (TGA Q500, TA Instruments Inc., Eden Prairie, MN, USA) at 20–600 °C with a heating rate of 10 °C/min in N_2_ atmosphere, and the equilibration time was 10 min.

### 3.7. Statistical Analysi

Three parallel experiments were conducted for each group of experiments, and the experimental data were expressed as the mean ± SD. The results were considered to differ significantly based on a significance level of *p* < 0.05.

## 4. Conclusions

In this study, based on blueberry anthocyanins, BAM and BAL were fabricated and characterized, and their preparation process was optimized. Their properties were systematically compared. The particle size, zeta potential, microtopography, and structure feature information of the BAM and BAL were compared. In addition, the preservation rates under both dark and light conditions were compared, and the thermal stabilities of the BAM and BAL were characterized. The results show that despite the simplicity of both preparation processes of the BAM and BAL, they both had high encapsulation efficiencies. The most important factors in the single factor experiment for microcapsule and liposome preparation were the content of CaCl_2_ and the amount of anthocyanin. Although no significant differences were found between the stability of the BAM and BAL, both could significantly maintain the light stability and thermodynamic stability of blueberry anthocyanins. This study of BAM and BAL provides a scientific recommendation for the wider application of blueberry anthocyanins as food and in other nutrition delivery fields.

## Figures and Tables

**Table 1:** Design and results of response surface method for microcapsule process optimization.

No.	Independent Variables			Response Value
	CaCl_2_ Concentration (%)	Sodium Alginate: CaCl_2_	Encapsulation Time (h)	Encapsulation Efficiency (%)
1	2	1:4	2	85.25
2	2	1:4	1	89.32
3	2	1:5	1.5	92.66
4	2	1:5	1.5	90.97
5	1.5	1:5	2	90.59
6	2	1:5	1.5	91.95
7	1.5	1:4	1.5	90.88
8	2.5	1:4	1.5	90.2
9	2	1:6	1	85.32
10	2.5	1:5	2	87.57
11	2	1:5	1.5	89.84
12	1.5	1:6	1.5	84.6
13	2.5	1:6	1.5	83.43
14	2	1:5	1.5	92.59
15	2.5	1:5	1	90.86
16	1.5	1:5	1	95.27
17	2	1:6	2	83.19

**Table 2:** Analysis of variance results for the quadratic polynomial model.

Source of Variation	Sum of Mean Square	Freedom	Mean Square	F-Value	*p*-Value
Models	184.78	9	20.53	8.55	0.0049
X_1_	12.20	1	12.20	5.08	0.0489
X_2_	45.51	1	45.51	18.94	0.0033
X_3_	23.12	1	23.12	9.62	0.0173
X_1_X_2_	0.060	1	0.060	0.025	0.8789
X_1_X_3_	0.99	1	0.99	0.41	0.5414
X_2_X_3_	0.91	1	0.91	0.38	0.5573
X_1_^2^	0.71	1	0.71	0.29	0.6039
X_2_^2^	94.39	1	94.39	39.29	0.0004
X_3_^2^	5	1	5	2.08	0.1923
Residuals	16.62	7	2.40		
Misfit term	11.10	3	3.70	2.59	0.1905
Error	5.72	4	1.43		
Total	201.60	16			
R^2^	0.9166				
Adj.R^2^	0.8093				

Note: *p* < 0.01, highly significant; 0.01 < *p* < 0.05, significant; *p* > 0.05, not significant.

**Table 3:** Design and results of response surface method for liposome process optimization.

No.	Independent Variables			Response Value
	Ultrasonic Time (min)	The Amount of Anthocyanins Added (mg)	Lecithin Addition (mg)	Encapsulation Efficiency (%)
1	8	10	120	75.25
2	8	10	80	73.32
3	8	12	100	82.99
4	8	12	100	83.97
5	6	12	120	69.59
6	8	12	100	80.59
7	6	10	100	76.88
8	10	10	100	87.20
9	8	14	80	67.35
10	10	1:5	120	70.70
11	8	1:5	100	79.78
12	6	1:6	100	63.67
13	10	1:6	100	69.23
14	8	1:5	100	78.58
15	10	1:5	80	74.26
16	6	1:5	80	77.21
17	6	14	120	63.16

**Table 4:** Analysis of variance (ANOVA) for the quadratic polynomial model.

Source of Variation	Sum of Mean Square	Freedom	Mean Square	F-Value	*p*-Value
Models	691.26	9	76.81	5.85	0.0148
X_1_	24.68	1	24.68	1.88	0.0129
X_2_	300.25	1	300.25	22.85	0.0020
X_3_	23.22	1	23.22	1.76	0.0263
X_1_X_2_	5.20	1	5.20	0.40	0.5493
X_1_X_3_	3.98	1	3.98	0.30	0.5992
X_2_X_3_	9.27	1	9.27	0.71	0.4287
X_1_^2^	15.42	1	15.42	1.17	0.3145
X_2_^2^	107.17	1	107.17	8.20	0.0232
X_3_^2^	174.17	1	174.17	13.25	0.0003
Residuals	91.98	7	13.14	-	-
Misfit term	72.40	3	24.13	4.93	0.0787
Error	19.56	4	1.43		
Total	763.24	16			
R^2^	0.9026				
Adj.R^2^	0.7993				

Note: *p* < 0.01, highly significant; 0.01 < *p* < 0.05, significant; *p* > 0.05, not significant.

**Table 5:** Factors and levels of response surface test for BAM.

Levels		Factors	
	CaCl_2_ Concentration (%)	Sodium Alginate: CaCl_2_	Encapsulation Time (h)
−1	1.5	1:4	1
0	2	1:5	1.5
1	2.5	1:6	2

**Table 6:** Factors and levels of response surface test for BAL.

Levels		Factors	
	Anthocyanins Concentration (mg)	Ultrasonic Time (min)	Soy Lecithin Concentration (mg)
−1	6	8	80
0	8	12	100
1	10	16	120

## Data Availability

The data presented in this study are available on request from the corresponding author.

## Sample Availability

Samples are not available from the authors.
